# Assessing the conflict-of-interest reporting practices in published health technology assessment reports

**DOI:** 10.1017/S0266462326104024

**Published:** 2026-07-22

**Authors:** Miro Vuković, Filipa Alpeza, Ana Marušić

**Affiliations:** 1 Center for Evidence-Based Medicine, University of Split School of Medicine, Croatia; 2 Center for Resilient Health, Stockholm School of Economics, Sweden; 3 Department of Research, University Hospital of Split, Spinčićeva 1, 21 000 Split, Croatia

**Keywords:** health technology assessment, conflict of interest, transparency, reporting standards, disclosure, governance, policy harmonization, EU Regulation 2021/2282

## Abstract

**Background:**

Health technology assessment (HTA) informs healthcare policy, reimbursement, and clinical decision making. Transparency in conflict-of-interest (CoI) reporting is critical to ensure the credibility and impartiality of HTA outputs. However, the extent and consistency of CoI disclosures across HTA reports remain unclear.

**Objectives:**

To systematically assess the frequency, form, and comprehensiveness of CoI reporting in published HTA reports and to identify possible patterns.

**Methods:**

We retrieved and screened 1,215 HTA records from the International HTA Database. After exclusions, 689 English-language reports were analyzed (full and mini HTAs (*n* = 254), rapid reviews (*n* = 166), and HTA research studies (*n* = 269)). CoI statements were categorized as positive (declared CoI) or negative (no CoI). Cross-country comparisons and content analyses of positive statements were conducted distinguishing between the presence of disclosure, its content and granularity, and the transparency of conflict management.

**Results:**

CoI statements were present in 71 percent of full/mini HTAs, 60 percent of rapid reviews, and 100 percent of HTA research studies. CoIs were declared in 14, 16, and 83 percent of these categories, respectively. Reporting practices varied widely among agencies, with few providing detailed or standardized disclosures. Financial CoI dominated, while nonfinancial and institutional CoI were less frequently addressed.

**Conclusions:**

CoI reporting in HTA remains inconsistent despite its recognized importance for transparency. Disclosure and management of conflicts represent distinct governance functions, both requiring attention. Our findings highlight a need for harmonized, individual-level disclosure policies, and a description of CoI management strategies in HTA processes.

## Introduction

Health technology assessment (HTA) is a systematic process that evaluates clinical, economic, social, and ethical implications of health technologies. HTA reports are intended to guide evidence-informed resource allocation in healthcare, thus affecting policy decisions and patient access to health interventions (1). Though HTA employs different methods compared to health research, it is widely acknowledged that principles of transparency and integrity are paramount for credible assessments. To that end, those involved in HTA are expected to declare potential conflicts of interest (CoI), which can arise when professional judgment regarding a primary interest, such as the strength and quality of evidence or patient welfare, is influenced by a secondary interest, such as financial or professional gain (2). In the context of HTA, undisclosed or poorly managed CoI could lead to biased assessments, potentially misguiding health reimbursement and regulatory decisions (Table 1). Moreover, the HTA process often involves multidisciplinary contributors, including clinicians, economists, patient representatives, and policy makers, each of whom may have different professional, institutional, or intellectual interests. The potential for nonfinancial and institutional CoI, such as academic allegiance or agency funding dependency, therefore warrants as much scrutiny as financial ties (3). To address the growing complexity of disclosure, several frameworks have been introduced. The International Committee of Medical Journal Editors (ICMJE) standardized disclosure form for biomedical publishing (4) and the European Network for Health Technology Assessment (EUnetHTA) Declaration of Interest and Confidentiality Undertaking (5) both seek to improve uniformity in CoI reporting. Furthermore, the Guidelines International Network (GIN) Principles for Disclosure of Interests and Management of Conflicts in Guidelines (6) provide a structured framework that addresses who should disclose, what should be disclosed, how conflicts should be classified and assessed, and how they should be managed. In January 2025, the European Union (EU) began implementing a new Regulation on Health Technology Assessment (EU 2021/2282), which creates a framework for collaborative and coordinated assessment of health technologies between EU Member States, and requires structured reporting and management of CoI. Despite the recognized importance of disclosing and addressing CoI, research across biomedical disciplines continues to reveal persistent inconsistencies in CoI reporting (7;8), and implementation of CoI guidelines across HTA agencies is fragmented. Previous analyses show that while some agencies, such as those in Northern and Western Europe, have adopted detailed CoI policies, others (particularly in emerging HTA systems) continue to rely on generic statements or lack formal requirements (9). However, the research in this area remains scarce and provides limited insights into the extent to which CoIs are disclosed, the types of interests recognized, and whether mitigation measures are described. Therefore, the aim of this study was to provide a descriptive comparative mapping of frequency and form of CoI reporting in internationally published HTA reports.

**Table 1.** Conflict of interest (CoI) disclosure in health technology assessment: key concepts Table 1. long description.

Purpose	To preserve the integrity and credibility of HTA outputs	
	To enable readers and decision makers to identify potential sources of bias	
	To distinguish between the presence of an interest and an active conflict	
	To promote accountability of HTA agencies and individual contributors	
Key dimensions	*Who should disclose?*	All individuals contributing to the assessment, including authors, external experts, reviewers, and patient representatives.
	*What should be disclosed?*	Financial interests (e.g., employment, grants, honoraria, and stocks), nonfinancial interests (e.g., intellectual conflicts and academic allegiances), and institutional interests (e.g., agency funding dependencies).
	*When to disclose?*	At the time of involvement, as well as in a specified timeframe in the past, and updated as circumstances change.
	*How managed?*	Through recusal from relevant deliberations, restricted participation, independent review, or balancing of conflicted and unconflicted expertise within assessment groups.
Importance of HTA outputs and decisions	Undisclosed or unmanaged CoI may bias evidence synthesis, assessment conclusions, and reimbursement recommendations.	
	Disclosure alone is insufficient; transparent management strategies are equally critical to ensure impartial decision making.	
	Conflicts may favor or disfavor a technology under assessment, highlighting the importance of bidirectional transparency.	

*Sources:* The information in the table was synthesized from established conflict of interest frameworks and published literature, including the GIN Principles for Disclosure of Interests and Management of Conflicts in Guidelines (6), the National Academies report on Conflict of Interest in Medical Research, Education, and Practice (2), the ICMJE disclosure framework (4), and supporting literature (3;16–19).

In this study, we use “conflict of interest” (CoI) as the primary term to describe situations where professional judgment may be influenced by secondary interests, while “competing interests” is retained when specifically referencing journal-based disclosures, consistent with ICMJE terminology (4). Critically, effective CoI governance encompasses not only disclosure – the act of transparently reporting interests – but also the management of declared conflicts through strategies such as recusal, restricted participation, or balancing of expertise within assessment groups (6;10). While there is no universal conceptualization of adequate CoI reporting, the abovementioned frameworks offer important guidance for practice. Given the substantial overlap between HTA and clinical guideline development in terms of evidence synthesis, expert involvement, and decision making (3;11;12), CoI practices in HTA should be understood within this broader ecosystem of evidence-based health decision making rather than as an isolated domain.

## Methods

### Data sourcing and retrieval

All records were retrieved from the International HTA database (available at https://database.inahta.org/) in October 2023. The International HTA database is an open-access global repository of bibliographic information about available HTAs commissioned or conducted by members of HTA organizations, including but not limited to the members of the International Network of Agencies for Health Technology Assessment (INAHTA). The search was performed using Python web scraping methods and included filters for publication type (full HTA, mini HTA, and rapid reviews; publication types described in Supplementary Table 1), language (English), and status of the project (completed). It should be noted that the International HTA Database has known limitations, including inconsistent classification of HTA outputs and broken or outdated links, which may affect the interpretation of findings and are discussed in detail in the limitations section.

A total of 1,215 records were imported into a spreadsheet, which was used to manage and analyze the data. The database provided information on the publication year, organization conducting the assessment, country of origin, and publication type. Publication types recognized by the database include “full HTA,” “mini HTA,” and “rapid review,” and their characteristics are described in Supplementary Table 1. This classification is done internally by institutions producing HTA products, based on the criteria outlined by the INAHTA (13).

### Record screening

Two reviewers (MV and FA) independently assessed each record for accessibility and eligibility. We excluded duplicates, records that were neither HTAs nor rapid reviews, texts written in languages other than English, and records that could not be opened due to URL issues.

### Data analysis

For all included records, the reviewers checked whether a CoI disclosure statement was present (yes/no). We extracted the statements wherever available and marked them as “Positive” (if interests were declared) or “Negative” (if none were declared). This approach allowed us to distinguish between the absence of disclosure (i.e., unknown status of CoI) and the absence of CoI. The data extraction matrix is available in Supplementary Table 2. Any misalignment between the reviewers was resolved through a research team discussion. Subsequently, we conducted a cross-country comparison and content analysis of positive statements, focusing on the nature of disclosed interests in each record and the style of reporting. While we used the ICMJE guidelines and insights from the background literature to guide this process, given the absence of an international gold standard CoI policy, our approach was iterative to ensure comprehensive coverage of diverse statements included in this study. Although we initially considered records to be either full/mini HTAs or rapid reviews, as indicated in the database, an additional possible classification became apparent during the analysis, which we referred to as “HTA research.” “HTA research” comprised records published in journals where completion of the ICMJE disclosure form is mandatory according to editorial guidelines. This category was analyzed separately and is not indicative of institutional HTA governance per se. Instead, we included it to serve as a benchmark case and to provide descriptive insights into the prevalence of positive statements in context of standardized and diligently enforced disclosure guidelines. Throughout the analysis, we noted and subsequently synthesized any other relevant observations and trends.

## Results

This study included 689 records (Supplementary Figure 1), the majority being HTA research (*n* = 269), followed by full and mini HTAs (*n* = 254) and rapid reviews (*n* = 166). While we refer to these categories for consistency, we note that the terminology used in the records was broader and varied by country. For instance, records were titled as Evidence Appraisal Reports (Wales, UK), Recommendations/Advice on Health Technologies (Scotland, UK), Horizon Scanning (Austria), Evidence Synthesis to Support a Generic Justification Decision (Ireland), Guide for Decision making (Spain), and Health Technology Reviews or Optimal Use Reports (Canada).

The largest group of excluded records was funding awards for HTA-related research (all from the United Kingdom), followed by research articles within the scope of health and social care delivery (all from the United Kingdom), non-English records, and resolutions on amendments of pharmaceutical directives (all from Germany). Publications such as student theses and executive summaries were beyond the scope of this study, and were labeled as “other” and excluded. The items published in languages other than English originated from Canada (*n* = 10), Sweden (*n* = 10), Norway (*n* = 9), Austria (*n* = 6), Spain (*n* = 4), Germany (*n* = 1), and Tunisia (*n* = 1). Some of the non-English records included courtesy translations; however, these did not capture CoI disclosure statements.

We noted that all excluded resolutions and health research were classified as “full HTAs” in the database. Additionally, we observed that certain records had dual classification, that is, they were listed among both HTAs and rapid reviews, explaining some of the duplicates in our sample.

A CoI disclosure statement was present in the majority of included full/mini HTAs and rapid reviews, and in all HTA research records. While the statements were predominantly “negative” across the HTAs and reviews, more than 80 percent of statements extracted from HTA research records reported some competing interests (Table 2).

**Table 2.** Overview of conflict of interest (CoI) reporting in the included records Table 2. long description.

	No. full/mini HTA (%)	No. rapid reviews (%)	No. supporting evidence studies/HTA research
Total	254	166	269
Had a CoI statement	180 (71%)	100 (60%)	269 (100%)
Statements with declared CoI^a^	25 (14%)	16 (16%)	222 (83%)
Described CoI management/justified involvement of experts with CoI^b^	1 (4%)	7 (44%)	NA

a Out of the total records that had a CoI statement.

b Out of statements with declared CoI.

### HTA research records

All records that we manually allocated to the HTA research category were classified as “full HTAs” by the database. However, these included three cohort studies from Taiwan, all with negative CoI statements, and 266 extended research articles conducted within the scope of the UK’s Health Technology Assessment program. The program, funded by the National Institute for Health Research (NIHR), supports evidence synthesis and primary research into the clinical, economic, and social impact of healthcare interventions (14). The NIHR studies were published in the *Health Technology Assessment* journal, with the most common study types being randomized controlled trials (*n* = 127) and systematic reviews (*n* = 79), often coupled with an economic evaluation. Completion of the ICMJE Disclosure of Interest form is a part of the journal’s submission requirements, and completed forms for all authors were publicly available. The presence of key terms from the ICMJE form in positive CoI statements is quantified in Table 3.

**Table 3.** Number of positive conflict of interest statements in National Institute for Health Research studies containing specified ICMJE terms^a^ Table 3. long description.

Key ICMJE terms	No. (%)
Grants/contracts	136 (61)
Royalties	10 (5)
Personal/consulting fees	102 (46)
Lectures	20 (9)
Travel support	19 (9)
Participation in relevant boards, committees, and panels	160 (72)
Patents	23 (10)
Stocks	15 (7)^b^
Nonfinancial support	35 (16)^c^

a Out of the total number of positive statements (*n* = 222); ICMJE – International Committee of Medical Journal Editors.

b In one instance, stocks belonged to the author’s family member.

c Refers to verbatim use of the term.

### Full/mini HTAs

Records in this category were published by institutions from 12 countries (counting Wales and Scotland separately) and by the EUnetHTA (Table 4). CoI reporting style was largely consistent within but varied substantially between institutions (see Supplementary Table 3 for detailed reporting practices per institution). In instances when institutions customized their reporting (i.e., did not use standardized forms or international guidelines), it was often unclear how CoI was defined. An exception to this was the Belgian Health Care Knowledge Centre, which had more detailed statements compared to the rest of the included institutions, and a relatively broad conceptualization of CoI, arguing that any stakeholder involved in a particular health topic by definition might have a certain degree of conflict of interest (direct quote from the organization: “All experts and stakeholders consulted within this report were selected because of their involvement in the topic of (subject of the HTA). Therefore, by definition, each of them might have a certain degree of conflict of interest to the main topic of this report.”).

**Table 4.** Overview of included agencies by country and Health Technology Assessment (HTA) report type Table 4. long description.

Country	Total reports	Full/mini HTAs	Rapid reviews	Institution(s)
Austria	85	82	3	Austrian Institute for Health Technology Assessment
Canada	74	54	20	Canadian Agency for Drugs and Technologies in Health (CADTH); WorkSafeBC; Institute of Health Economics
International (EUnetHTA)	62	1	61	European Network for Health Technology Assessment
Wales (UK)	43	1	42	Health Technology Wales
Malaysia	38	38	0	Malaysian Health Technology Assessment
Norway	28	27	1	Norwegian Institute of Public Health
Ireland	28	14	14	Health Information and Quality Authority
Belgium	20	11	9	Belgian Health Care Knowledge Centre
Switzerland	19	16	3	Swiss Federal Office of Public Health
United States	13	1	12	Institute for Clinical and Economic Review; Agency for Healthcare Research and Quality (AHRQ)
Australia	5	5	0	Adelaide Health Technology Assessment
Scotland (UK)	3	3	0	Scottish Health Technologies Group
Spain	2	1	1	Andalusian Health Technology Assessment Area; Basque Office for Health Technology Assessment
Total	420	254	166	

### Rapid reviews

Most records in this category were authored by the EUnetHTA. Similar to full/mini HTAs, CoI reporting style was heterogeneous, with some institutions following international guidelines and others adopting a customized approach (see Supplementary Table 4 for detailed reporting practices per institution). In reviews from Norway and Wales, authors considered potential conflicts of interest in included studies but did not seem to disclose their own competing interests.

### Content analysis of positive CoI statements in full/mini HTAs and rapid reviews

Forty-one HTAs and rapid reviews had positive CoI statements (Supplementary Table 5). In 66 percent of these (*n* = 27), more than one person involved in producing the record had declared CoI. Table 5 summarizes the types of CoIs and their frequency. The greatest number of records with positive statements came from the Belgian Health Care Knowledge Centre (*n* = 17), possibly due to the Centre’s elaborate classification and broad definition of CoI. The remaining records originated from Canada (*n* = 9), Ireland (*n* = 3), Norway (*n* = 2), the United States (*n* = 1), Austria (*n* = 1), and Spain (*n* = 1), alongside seven records published by the EUnetHTA. Despite adopting a detailed approach to declaring potential interests, Belgian records did not justify the involvement of experts with known CoIs, as was the case in most other countries. The nonmention of CoI management strategies made it uncertain whether and how integrity was ensured. Exceptions to this were the statements by the EUnetHTA, alongside one Norwegian record. EUnetHTA managed the presence of CoI by, for instance, allowing experts to comment on the draft assessment without having access to any potentially confidential material, or by allowing experts to provide input but excluding them from decision making. The exact year when activities described in CoI statements occurred, such as subsidized travel, paid speaking engagements, or receipt of research funding, was specified in only ten records.

**Table 5.** Nature of declared conflicts of interest (CoI) in Health Technology Assessment (HTA) and rapid reviews (*n* = 41) Table 5. long description.

Categories of CoI	Description	Frequency, no. (%)	Examples^a^
Participation in a stakeholder group that may be affected by results^b^	This may include being a member or holding a particular function in relevant organizations, associations, panels, societies, and similar. This may or may not be salaried.	20 (49%)	*Dr. X serves in a leadership role at a network of centers of excellence.* *One external expert, Dr. Y, is president of a professional society.*
Leading or being a member of a research group	Participation in research relevant to the health technology under assessment.	21 (51%)	*XX and YY declared to have acted as principal investigators in a prospective cohort study (health technology under assessment) which was funded by industry.* *ZZ reports being employed as a postdoc in a research project that will investigate two treatments for (condition affected by the outcomes of the assessment).*
Receiving research support	Receipt of grants, fees, materials, or other support for the conduct of research. Also includes awards such as fellowships and participation in industry-funded research.	20 (49%)	*Dr. X has accepted speaking engagements and has received research funding from industry.* *YY received funding from a university institute to conduct a pilot clinical trial of a medication for (condition affected by the outcomes of the assessment).*
Paid engagements with an entity that may be impacted by results	Receipt of salary, honoraria, consulting fees, and similar from relevant companies/organizations.	24 (59%)	*Dr. X has been employed by the company (member of the board of directors), which produces devices for a type of therapy relevant to the assessment.* *Dr. Y declared having received funding from several companies within five years preceding this project. Funding was received for advisory work, as well as educational and research activities.*
Support for travel, training, speaking engagements, and other events	Receipt of payments to attend or take part in training courses, conferences, and similar events.	22 (54%)	*The co-authors X and Y received honoraria from the industry for speaking at symposia and were Advisory Board Members for the industry.* *Professor Z has attended symposia related to and gave lectures on the topic of (a procedure relevant to the assessment), which were sponsored by the industry.*
Intellectual property	Owning IP rights relevant to the health technology being assessed.	5 (12%)	*Holder of intellectual property (patent, product developer, copyrights, trademarks, etc.): XX (inventors certificate), YY (patent)* *Holder of intellectual property (patent, product developer, copyrights, trademarks, etc.): ZZ (patent for a technology relevant to the assessment)*
Other	Any declared conflict of interest beyond the scope of the remaining categories.	8 (20%)	*XX reports being a clinician and thus may have a personal preference for a certain type of treatment.* *YY: a chair in my research group was partially funded by industry.*
Support for publications	Receiving funds to write a manuscript, report, or similar piece of work.	10 (24%)	*Dr. XX has received funding from industry for research, being on a user’s group panel, writing articles or editorials, and being on an expert panel.* *Dr. YY received funding/honorariums for writing a chapter concerning (a health issue relevant to the assessment) for an international organization.*
Stocks and shares	Owning capital, stocks, or shares of relevance to the health technology being assessed.	6 (15%)	*XX disclosed a financial interest (i.e., stocks) in industry.* *Owner of subscribed capital, options, shares, or other financial instruments: YY (private company).*

a Statements were anonymized.

b It was often unclear whether this was remunerated.

## Discussion

### Overall interpretation and context

To the best of our knowledge, this study represents the first multi-agency, cross-sectional analysis of CoI reporting practices within publicly available HTA reports. Across 689 included records retrieved from the International HTA Database, we identified substantial heterogeneity in both the presence and depth of CoI disclosures across agencies, countries, and HTA product types. While most full and mini HTAs, as well as rapid reviews, included a CoI statement, detailed and individual-level disclosures were uncommon, and reporting was frequently limited to generic or collective declarations. Moreover, only EUnetHTA records described how the bias that may arise from involving assessors with declared CoI was mitigated. This variability is consistent with previous observations that governance structures and transparency norms in HTA remain unevenly developed across jurisdictions (15–17). When evaluated against the GIN principles for disclosure and management (6), this heterogeneity reflects not merely variation in practice but substantive deficiencies in several dimensions of CoI governance: many agencies lacked structured disclosure requirements, few addressed nonfinancial or intellectual conflicts, and management of declared interests was rarely discussed.

HTA research studies published in peer-reviewed journals demonstrated markedly higher disclosure completeness, with all such studies including CoI statements and over 80 percent reporting competing interests. This pattern appears to be driven primarily by mandatory journal submission requirements, most notably completion of the ICMJE disclosure form, rather than by national HTA policies. Similar effects of editorial requirements on disclosure practices have been documented in biomedical research and guideline development, suggesting that external enforcement mechanisms may in some settings be more stringent than CoI governance within HTA agencies (6;11). Nevertheless, the ICMJE framework should be understood as an example of a viable disclosure mechanism rather than as an implicit benchmark for HTA agencies, as it primarily addresses financial conflicts and may not capture the full range of interests relevant to HTA decision making, including intellectual conflicts or indirect financial interests such as those arising from clinical practice involvement (18).

Across HTAs and rapid reviews, financial interests were the most frequently declared conflicts, reflecting their relative ease of identification and reporting. In contrast, nonfinancial, intellectual, and institutional conflicts were less consistently recognized. Many CoI statements lacked critical contextual detail, particularly regarding whether declared roles, such as participation in advisory boards or expert groups, were remunerated. This ambiguity blurs the distinction between financial and nonfinancial interests and mirrors concerns raised in broader CoI literature about the limited interpretability of incomplete disclosures (3). Only a small number of agencies explicitly described how declared conflicts were managed. The absence of such information limits readers’ ability to assess whether disclosed conflicts were meaningfully addressed and reinforces prior critiques that disclosure alone is insufficient without transparent conflict management (10;12;18). Together, these findings suggest that the mere presence of a disclosure statement should not be equated with effective CoI governance, with its practical value constrained by vague disclosures and ambiguity around how reported CoIs were subsequently handled. Moreover, the directionality of declared conflicts received limited attention; conflicts may not only favor but also disfavor a product under assessment, for instance, when an assessor has ties to a competing product or holds a preexisting negative view. Recognizing this bidirectionality is important for transparent interpretation (19). Furthermore, in specialized areas such as rare disease technologies, where finding experts without any CoI is particularly challenging, some HTA bodies have attempted to balance conflicts within expert groups rather than exclude all conflicted individuals. The extent to which such balancing strategies are employed, and whether they are transparent to readers, warrants further attention.

These observations underscore the need to holistically address the multiple dimensions of CoI governance: (a) presence of disclosure, (b) content and granularity of disclosure, (c) type of disclosed interests using established frameworks such as the GIN principles (6), and (d) transparency of conflict management strategies.

### Implications and recommendations for future practice

Taken together, these findings highlight several recurring shortcomings in current CoI reporting practices that have direct implications for HTA governance. The substantially higher completeness observed in journal-published HTAs suggests that adoption of standardized, internationally recognized disclosure templates could enhance consistency and clarity if implemented within institutional HTA settings. Harmonization of disclosure formats across agencies may also facilitate cross-country comparison and reuse of HTA outputs, an increasingly important objective in the context of European HTA collaboration (20). Importantly, the unstandardized disclosure practices also blur the lines between heterogeneity and deficiency in CoI reporting. At the same time, the frequent absence of information on how declared conflicts are assessed or managed indicates a need for greater transparency regarding conflict mitigation, and demonstrates that CoI disclosure should not be viewed as a proxy for effective CoI governance. Instead, explicit reporting of management strategies would strengthen accountability and help distinguish between disclosed but unmanaged conflicts and those actively addressed through procedural safeguards. Interpretive challenges were also linked to structural features of the International HTA Database. Inconsistent classification of HTA outputs, combined with incomplete metadata and access barriers such as broken or outdated links, limited comparability and reuse of existing assessments. Addressing these database-related issues, through clearer categorization criteria, improved maintenance, and more robust archiving practices, could reduce duplication of work and support more efficient knowledge sharing. Finally, language-related barriers remain a significant obstacle. Several non-English HTA reports and courtesy translations contained abbreviated or missing CoI statements, reducing transparency for international audiences. More systematic approaches to translation and dissemination that preserve the integrity of CoI disclosures would improve accessibility and usability beyond national contexts, as well as reduce the burden of work duplication. An important interpretive distinction that emerges from these findings is between the absence of CoI disclosure and the actual absence of conflicts. Generic negative statements do not necessarily indicate that no conflicts exist; rather, they may reflect narrow definitions of what constitutes a conflict or insufficient inquiry into potential interests. Moreover, CoI disclosure in and of itself may be insufficient to minimize the risk of biased assessment, and should be accompanied by a clear description of approaches put in place to preserve impartiality.

### Future research recommendations

Future research should move beyond descriptive assessments of CoI reporting toward evaluating the consequences of differing disclosure practices. In particular, studies examining whether the quality or completeness of CoI reporting is associated with HTA conclusions, recommendations, or reimbursement outcomes would provide valuable insight into the real-world implications of incomplete disclosure. Further work is also needed to explore how conflicts are managed in practice. Qualitative research involving HTA agencies could clarify how CoI policies are operationalized, how mitigation decisions are made, and how agencies balance the need for expert input with independence. Developing clearer operational definitions for nonfinancial and institutional conflicts, especially in cases where remuneration is unclear, would further improve consistency and interpretability. Applying established frameworks such as the GIN principles (6) as analytic benchmarks in future evaluations could provide a more systematic basis for assessing the adequacy of CoI governance across agencies. Research should also investigate the role of intellectual conflicts and indirect financial interests – such as those arising from clinical practice, institutional affiliations, or involvement in competing products – which remain underexplored in the HTA context. Finally, future studies should examine structural determinants of transparency, including HTA system maturity, funding models, and language practices. Understanding how these factors shape CoI reporting may contribute to strengthening HTA processes and facilitating cross-border collaboration in conducting assessments.

### Strengths and limitations

The principal strength of this study lies in its international scope and systematic approach, enabling cross-sectional comparison of CoI reporting practices among diverse HTA bodies. Dual-reviewer screening and iterative coding enhanced internal validity. However, several limitations warrant consideration. Classification of HTA outputs within the International HTA Database appears inconsistently applied, as designations are largely self-assigned by submitting organizations, introducing potential misclassification bias. These database-related limitations, including inconsistent classification and broken links, affect the comparability and interpretation of findings across agencies. Restriction to English-language reports may have led to an underestimation of transparency in regions where HTAs are published in national languages. This limitation is noteworthy in the context of CoI research, as transparency norms, disclosure requirements, and the very conceptualization of conflicts may differ significantly across linguistic and cultural contexts. The exclusion of non-English reports may thus obscure important regional practices that could inform international harmonization efforts. In addition, some potentially eligible reports could not be accessed due to broken or outdated links, underscoring weaknesses in current dissemination and archiving practices. Finally, the analysis relied solely on publicly available documentation and may not reflect internal disclosure mechanisms maintained by individual agencies.

## Conclusions

CoI reporting within HTA remains variable, with notable regional and institutional differences. While some agencies have implemented robust systems or adopted international reporting guidelines, others continue to rely on minimal or generic statements. The absence of consistent detail, particularly regarding remuneration and conflict management, limits interpretability and may constrain public accountability. These findings carry policy implications: transparency of HTA processes could potentially be enhanced by aligning institutional reporting practices with established frameworks such as the GIN principles (6), standardizing both disclosure and management procedures, and addressing language and accessibility barriers. Future policy efforts should recognize that disclosure alone is insufficient and that transparent conflict management is equally essential for the credibility of HTA outputs.

## Supporting information

10.1017/S0266462326104024.sm001 Vuković et al. supplementary material Vuković et al. supplementary material

## Supplementary material

The supplementary material for this article can be found at http://doi.org/10.1017/S0266462326104024.

## Table 1. Long description

This table presents key concepts of conflict of interest (CoI) disclosure in health technology assessment (HTA), organised into three sections: Purpose, Key dimensions, and Importance for HTA outputs and decisions. The Purpose section lists four aims of CoI disclosure: to preserve the integrity and credibility of HTA outputs; to enable readers and decision-makers to identify potential sources of bias; to distinguish between the presence of an interest and an active conflict; and to promote accountability of HTA agencies and individual contributors. The Key dimensions section answers four questions. Who should disclose: all individuals contributing to the assessment, including authors, external experts, reviewers, and patient representatives. What should be disclosed: financial interests (for example, employment, grants, honoraria, stocks), non-financial interests (for example, intellectual conflicts, academic allegiances), and institutional interests (for example, agency funding dependencies). When to disclose: at the time of involvement as well as in a specified timeframe in the past, and updated as circumstances change. How managed: through recusal from relevant deliberations, restricted participation, independent review, or balancing of conflicted and unconflicted expertise within assessment groups. The section on importance for HTA outputs and decisions makes three points: undisclosed or unmanaged CoI may bias evidence synthesis, assessment conclusions, and reimbursement recommendations; disclosure alone is insufficient, as transparent management strategies are equally critical to ensure impartial decision-making; and conflicts may favour or disfavour a technology under assessment, highlighting the importance of bidirectional transparency. A source note states that the information in the table was synthesised from established conflict of interest frameworks and published literature, including the GIN Principles for Disclosure of Interests and Management of Conflicts in Guidelines, the National Academies report on Conflict of Interest in Medical Research, Education, and Practice, the ICMJE disclosure framework, and supporting literature.

Navigate back to Table 1..

## Table 2. Long description

This table summarises conflict of interest (CoI) reporting across the three categories of included records: full/mini health technology assessments (HTAs), rapid reviews, and supporting evidence studies (HTA research). Of 254 full/mini HTAs, 180 (71%) had a CoI statement; of these statements, 25 (14%) declared a CoI, and of those, 1 (4%) described CoI management or justified the involvement of experts with CoI. Of 166 rapid reviews, 100 (60%) had a CoI statement; 16 of these statements (16%) declared a CoI, and 7 of those (44%) described CoI management or justified expert involvement. Of 269 HTA research records, all 269 (100%) had a CoI statement and 222 (83%) declared a CoI; the CoI management row was not applicable to this category. Table notes specify that percentages for statements with declared CoI are calculated out of the total records that had a CoI statement, and percentages for described CoI management are calculated out of the statements with declared CoI.

Navigate back to Table 2..

## Table 3. Long description

This table lists key terms from the International Committee of Medical Journal Editors (ICMJE) disclosure form and the number and percentage of positive conflict of interest statements in National Institute for Health Research studies containing each term, out of the total of 222 positive statements. Grants or contracts appeared in 136 statements (61%); royalties in 10 (5%); personal or consulting fees in 102 (46%); lectures in 20 (9%); travel support in 19 (9%); participation in relevant boards, committees and panels in 160 (72%); patents in 23 (10%); stocks in 15 (7%), where in one instance the stocks belonged to the author’s family member; and non-financial support in 35 (16%), referring to verbatim use of the term.

Navigate back to Table 3..

## Table 4. Long description

This table lists the countries whose agencies produced the included records, with the total number of reports, the split between full/mini health technology assessments (HTAs) and rapid reviews, and the producing institutions. Austria: 85 reports (82 full/mini HTAs, 3 rapid reviews), Austrian Institute for Health Technology Assessment. Canada: 74 reports (54 and 20), Canadian Agency for Drugs and Technologies in Health (CADTH), WorkSafeBC, and Institute of Health Economics. International (EUnetHTA): 62 reports (1 and 61), European Network for Health Technology Assessment. Wales (UK): 43 reports (1 and 42), Health Technology Wales. Malaysia: 38 reports (38 and 0), Malaysian Health Technology Assessment. Norway: 28 reports (27 and 1), Norwegian Institute of Public Health. Ireland: 28 reports (14 and 14), Health Information and Quality Authority. Belgium: 20 reports (11 and 9), Belgian Health Care Knowledge Centre. Switzerland: 19 reports (16 and 3), Swiss Federal Office of Public Health. United States: 13 reports (1 and 12), Institute for Clinical and Economic Review and Agency for Healthcare Research and Quality (AHRQ). Australia: 5 reports (5 and 0), Adelaide Health Technology Assessment. Scotland (UK): 3 reports (3 and 0), Scottish Health Technologies Group. Spain: 2 reports (1 and 1), Andalusian Health Technology Assessment Area and Basque Office for Health Technology Assessment. In total, there were 420 reports: 254 full/mini HTAs and 166 rapid reviews.

Navigate back to Table 4..

## Table 5. Long description

This table describes nine categories of declared conflicts of interest (CoI) identified in the 41 health technology assessments and rapid reviews with positive CoI statements. For each category, it gives a description, the number and percentage of records in which it appeared, and anonymised examples; categories are not mutually exclusive, as one record could declare several types of CoI (1). Participation in a stakeholder group that may be affected by results, 20 records (49%): being a member of or holding a function in relevant organisations, associations, panels or societies, which may or may not be salaried; it was often unclear whether this was remunerated. Examples: a doctor serving in a leadership role at a network of centres of excellence; an external expert who is president of a professional society (2). Leading or being a member of a research group, 21 records (51%): participation in research relevant to the health technology under assessment. Examples: authors acting as principal investigators in an industry-funded prospective cohort study on the assessed technology; an author employed as a postdoc in a project investigating treatments for the condition affected by the assessment (3). Receiving research support, 20 records (49%): receipt of grants, fees, materials or other support for the conduct of research, including fellowships and participation in industry-funded research. Examples: a doctor who accepted speaking engagements and received research funding from industry; an author funded by a university institute to conduct a pilot clinical trial of a relevant medication (4). Paid engagements with an entity that may be impacted by results, 24 records (59%): receipt of salary, honoraria, consulting fees and similar from relevant companies or organisations. Examples: a doctor employed as a board member by a company producing devices for a therapy relevant to the assessment; a doctor who received funding from several companies for advisory, educational and research activities within five years preceding the project (5). Support for travel, training, speaking engagements and other events, 22 records (54%): payments to attend or take part in training courses, conferences and similar events. Examples: co-authors who received industry honoraria for speaking at symposia and served as advisory board members; a professor who attended and lectured at industry-sponsored symposia on a procedure relevant to the assessment (7). Intellectual property, 5 records (12%): owning intellectual property rights relevant to the assessed technology, such as patents, product development, copyrights or trademarks. Examples: holders of an inventor’s certificate or a patent for a technology relevant to the assessment (8). Other, 8 records (20%): any declared CoI beyond the remaining categories. Examples: a clinician noting a possible personal preference for a certain type of treatment; a research group chair partially funded by industry (9). Support for publications, 10 records (24%): receiving funds to write a manuscript, report or similar work. Examples: a doctor funded by industry for research, panel membership and writing articles or editorials; a doctor who received honoraria for writing a chapter on a relevant health issue for an international organisation (13). Stocks and shares, 6 records (15%): owning capital, stocks or shares of relevance to the assessed technology. Examples: an author with disclosed stocks in industry; an owner of subscribed capital or financial instruments in a private company. A table note states that all statements were anonymised.

Navigate back to Table 5..
