# Supplementary material for: Assessing the conflict-of-interest reporting practices in published health technology assessment reports
Source: Int J Technol Assess Health Care. 2026 Jul 22;42(1):e75. doi: 10.1017/S0266462326104024 (PMC13735833; doi:10.1017/S0266462326104024)
Supplement: Supplementary file 1 [file S0266462326104024sup001.docx]

**Supplementary Table 1. Definitions for types of records in the International HTA Database, developed by the Quality Assurance Group of INAHTA**

|  | *Always* | *Optionally* |
| --- | --- | --- |
| **Full HTA** | - describe the characteristics and current use of the technology;  - evaluate safety and effectiveness issues;  - determine the cost-effectiveness of the technology (e.g., through economic modelling, when it is appropriate);  - provide information on costs/financial impact;  - discuss organisational considerations;  - conduct a comprehensive systematic literature review or a systematic review of high-level evidence;  - critically appraise the quality of the evidence base. | - address ethical, social, and legal considerations. |
| **Mini HTA** | - describe the characteristics and current use of the technology;  - evaluate safety and effectiveness issues;  - provide information on costs/financial impact;  - conduct a comprehensive systematic literature review or a systematic review of high-level evidence;  - critically appraise the quality of the evidence base. | - address organisational considerations. |
| **Rapid Review** | - describe the characteristics and current use of the technology, and  - Evaluate safety and effectiveness issues | - (often) conduct a review of only high-level evidence or of recent evidence, and may restrict the literature search to one or two databases  - Critically appraise the quality of the evidence base  - Provide information on costs/financial impact |

Taken verbatim from: http://www.inahta.org/hta-tools-resources/ipt-marks/

**Supplementary Table 2. Data extraction matrix**

| Publication link | Publication type | Publication year | Organisation | Country | Conflict of interest statement (yes/no) | CoI statement (verbatim) | CoI (positive/negative) | Type of CoI |
| --- | --- | --- | --- | --- | --- | --- | --- | --- |
|  |  |  |  |  |  |  |  |  |

**Supplementary Table 3. Overview of how conflicts of interest were reported in included full/mini HTAs** (n=254)

| **Featured countries** | **Number of reports** | **Institution(s)** | **Comments on CoI statements and reporting** |
| --- | --- | --- | --- |
| Austria | 82 | Austrian Institute for Health Technology Assessment *(formerly Ludwig Boltzmann Institute for Health Technology Assessment, LBI)* | All reports from the Austrian Institute included a CoI statement, which was based on the ICMJE form.  For example:  *‘All authors and the reviewers involved in the production of this report have declared they have no conflicts of interest in relation to the technology assessed according to the Uniform Requirements of Manuscripts Statement of Medical Journal Editors (www.icmje.org).’*  Reports from LBI occasionally referred to the EUnetHTA Declaration of Interest and Confidentiality Undertaking statement or lacked a CoI statement. |
| Canada | 54 | Canadian Agency for Drugs and Technologies in Health (CADTH) | The majority of CADTH reports had a CoI statement, which was customised. |
|  |  | WorkSafeBC | None of the WorkSafeBC records had a CoI statement. |
|  |  | Institute of Health Economics | The Institute of Health Economics considered competing interests to be “*financial interest or non-financial interest, either direct or indirect, that would affect the research contained in this report or create a situation in which a person’s judgement could be unduly influenced by a secondary interest, such as personal advancement.”* |
| Malaysia | 38 | Malaysian Health Technology Assessment | All records had a consistent CoI statement: “*The author of this report has no competing interest in this subject and the preparation of this report is entirely funded by the Ministry of Health Malaysia.”* |
| Norway | 27 | Norwegian Institute of Public Health | CoI statements were customised and not always reported.  For example:  *“The authors certify that they have no affiliations with or involvement in any organisation or entity with any financial or non-financial interest in the subject matter or materials discussed in this review. In addition, consulted experts have declared no conflict of interest.”* |
| Switzerland | 16 | Swiss Federal Office of Public Health | CoI statements were customised and not always reported.  Example: “*The authors have no financial, academic, personal or any other conflicts of interest to declare in relation to this project.”* |
| Ireland | 14 | Health Information and Quality Authority | CoI statements were customised and consistently reported.  Example: “*There were no reported conflicts of interest for the Expert Advisory Group or Evaluation Team.”* |
| Belgium | 11 | Belgian Health Care Knowledge Centre | CoI statements were customised and consistently reported. All statements were positive. |
| Australia | 5 | Adelaide Health Technology Assessment | Only one record had a CoI statement, which briefly reported:  *‘There were no conflicts of interest.’* |
| Scotland (UK) | 3 | Scottish Health Technologies Group (SHTG) | Records from SHTG had a custom CoI statement, albeit vague. For example: *‘Declarations of interest were received from all experts.’* |
| United States | 1 | Institute for Clinical and Economic Review | The CoI statement specified that financial interests greater than $5,000 must be declared. |
| - | 1 | European Network for Health Technology Assessment | Standardised EUnetHTA statement was consistently reported:  *“All authors, co-authors, dedicated reviewers, external experts (health care professionals, patients or patient representatives) involved in the production of this assessment have declared they have no conflicts of interest in relation to the technology and comparator(s) assessed according to the EUnetHTA declaration of interest (DOI) form, which was evaluated following the EUnetHTA Procedure Guidance for handling DOI form (*[*https://eunethta.eu/doi)*](https://eunethta.eu/doi))*.”* |
| Wales (UK) | 1 | Health Technology Wales | The CoI statement was based on the EUnetHTA form. |
| Spain | 1 | Andalusian Health Technology Assessment Area | The CoI statement was absent. |

**Supplementary Table 4. Overview of how conflicts of interest were reported in the included rapid reviews** (n=166)

| **Featured countries** | **Number of features** | **Institution(s)** | **Comments on CoI statements and reporting** |
| --- | --- | --- | --- |
| NA | 61 | EUnetHTA | Standardised EUnetHTA statement was consistently reported:  *“All authors and co-authors involved in the production of this living document have declared they have no conflicts of interest in relation to the technology and comparator(s) assessed according to the EUnetHTA declaration of interest (DOI) form. Conflict of Interest was evaluated following the EUnetHTA Procedure Guidance for handling DOI form (*[*https://eunethta.eu/doi)*](https://eunethta.eu/doi))*.”* |
| Wales (UK) | 42 | Health Technology Wales | The CoI statement was absent in all but one record:  *“A range of clinical experts from the UK commented on drafts of this report, reporting any declarations of interest in advance. Their views were documented and have been actioned accordingly.”* |
| Canada | 20 | Canadian Agency for Drugs and Technologies in Health | The CoI statement was absent. |
| Ireland | 14 | Health Information and Quality Authority | A customised CoI statement was included in some but not all reviews.  Example: *‘No potential conflicts of interest were declared by members of the Expert Advisory Group.”* |
| United States | 12 | Agency for Healthcare Research and Quality | The CoI statement was consistently reported and specified that financial interests greater than $5,000 must be declared.  Example: “*None of the investigators have any affiliations or financial involvement that conflicts with the material presented in this report.”*  The rationale for involving individuals who declare competing interests was also provided: “*Because of their unique clinical or content expertise, individuals with potential nonfinancial conflicts may be retained.2* |
| Belgium | 9 | Belgian Health Care Knowledge Centre | The CoI statement was customised and consistently reported. It was noted that “*All members of the KCE Expert Team make yearly declarations of interest and further details of these are available upon request.”* |
| Austria | 3 | Austrian Institute for Health Technology Assessment | The CoI statement was consistently reported and based on the ICMJE form:  *“All authors involved in the production of this report have declared they have no conflicts of interest in relation to the technology assessed according to the Uniform Requirements of Manuscripts Statement of Medical Journal Editors (*[*www.icmje.org)*](http://www.icmje.org))*.”* |
| Switzerland | 3 | Swiss Federal Office of Public Health | The CoI statement was customised and present in two of the included reviews:  *“None of the individuals involved in preparing this report have material, professional, familial, or financial affiliations that create actual or potential conflicts of interest related to the preparation of this report.*” |
| Spain | 1 | Basque Office for Health Technology Assessment | *“The authors declare that they have no conflict of interest in relation to this report.”* |
| Norway | 1 | Norwegian Institute of Public Health | The CoI statement was absent. |

**Supplementary Table 5. Types of conflicts of interest among reports with disclosed conflicts of interest**

| **Source** | **Database classification** | **Year** | **Origin** | **1** | **2** | **3** | **4** | **5** | **6** | **7** | **8** | **9** |
| --- | --- | --- | --- | --- | --- | --- | --- | --- | --- | --- | --- | --- |
| 1 | Rapid Review | 2018 | NA (EUNetHTA) |  |  |  |  |  |  |  |  |  |
| 2 | Rapid Review | 2017 | NA (EUNetHTA) |  |  |  |  |  |  |  |  |  |
| 3 | Rapid Review | 2019 | NA (EUNetHTA) |  |  |  |  |  |  |  |  |  |
| 4 | Rapid Review | 2018 | NA (EUNetHTA) |  |  |  |  |  |  |  |  |  |
| 5 | Rapid Review | 2019 | NA (EUNetHTA) |  |  |  |  |  |  |  |  |  |
| 6 | Rapid Review | 2017 | NA (EUNetHTA) |  |  |  |  |  |  |  |  |  |
| 7 | Rapid Review | 2018 | NA (EUNetHTA) |  |  |  |  |  |  |  |  |  |
| 8 | Full HTA | 2021 | Austria |  |  |  |  |  |  |  |  |  |
| 9 | Rapid Review | 2023 | Belgium |  |  |  |  |  |  |  |  |  |
| 10 | Rapid Review | 2015 | Belgium |  |  |  |  |  |  |  |  |  |
| 11 | Rapid Review | 2015 | Belgium |  |  |  |  |  |  |  |  |  |
| 12 | Rapid Review | 2015 | Belgium |  |  |  |  |  |  |  |  |  |
| 13 | Rapid Review | 2015 | Belgium |  |  |  |  |  |  |  |  |  |
| 14 | Rapid Review | 2019 | Belgium |  |  |  |  |  |  |  |  |  |
| 15 | Rapid Review | 2021 | Belgium |  |  |  |  |  |  |  |  |  |
| 16 | Full HTA | 2015 | Belgium |  |  |  |  |  |  |  |  |  |
| 17 | Full HTA | 2015 | Belgium |  |  |  |  |  |  |  |  |  |
| 18 | Full HTA | 2018 | Belgium |  |  |  |  |  |  |  |  |  |
| 19 | Full HTA | 2019 | Belgium |  |  |  |  |  |  |  |  |  |
| 20 | Full HTA | 2019 | Belgium |  |  |  |  |  |  |  |  |  |
| 21 | Full HTA | 2019 | Belgium |  |  |  |  |  |  |  |  |  |
| 22 | Full HTA | 2019 | Belgium |  |  |  |  |  |  |  |  |  |
| 23 | Full HTA | 2020 | Belgium |  |  |  |  |  |  |  |  |  |
| 24 | Full HTA | 2022 | Belgium |  |  |  |  |  |  |  |  |  |
| 25 | Full HTA | 2023 | Belgium |  |  |  |  |  |  |  |  |  |
| 26 | Full HTA | 2019 | Canada |  |  |  |  |  |  |  |  |  |
| 27 | Full HTA | 2019 | Canada |  |  |  |  |  |  |  |  |  |
| 28 | Full HTA | 2021 | Canada |  |  |  |  |  |  |  |  |  |
| 29 | Full HTA | 2018 | Canada |  |  |  |  |  |  |  |  |  |
| 30 | Full HTA | 2017 | Canada |  |  |  |  |  |  |  |  |  |
| 31 | Full HTA | 2019 | Canada |  |  |  |  |  |  |  |  |  |
| 32 | Full HTA | 2016 | Canada |  |  |  |  |  |  |  |  |  |
| 33 | Full HTA | 2022 | Canada |  |  |  |  |  |  |  |  |  |
| 34 | Mini HTA | 2021 | Canada |  |  |  |  |  |  |  |  |  |
| 35 | Rapid Review | 2021 | Ireland |  |  |  |  |  |  |  |  |  |
| 36 | Rapid Review | 2023 | Ireland |  |  |  |  |  |  |  |  |  |
| 37 | Mini HTA | 2023 | Ireland |  |  |  |  |  |  |  |  |  |
| 38 | Full HTA | 2019 | Norway |  |  |  |  |  |  |  |  |  |
| 39 | Full HTA | 2021 | Norway |  |  |  |  |  |  |  |  |  |
| 40 | Full HTA | 2016 | Spain |  |  |  |  |  |  |  |  |  |
| 41 | Full HTA | 2022 | United States |  |  |  |  |  |  |  |  |  |

Types of conflict of interest:

1: Participation in a stakeholder group that may be affected by the results

2: Leading or being a member of a research group

3: Receiving research support

4: Paid engagements with an entity that may be impacted by results

5: Support for travel, training, speaking engagements, and other events

6: Intellectual property

7: Other

8: Support for publications

9: Stocks and shares

List of reports

1. https://eunethta.eu/final-assessment-report-on-mammaprint-added-value-of-using-the-gene-expression-signature-test-mammaprint-for-adjuvant-chemotherapy-decision-making-in-early-breast-cancer/
2. https://eunethta.eu/final-assessment-report-on-midostaurin-rydapt-in-combination-with-standard-daunorubicin-and-cytarabine-induction-and-high-dose-cytarabine-consolidation-chemotherapy/
3. https://eunethta.eu/octa20-final-assessment-report-is-now-available-2/
4. https://eunethta.eu/otca11-custom-made-or-customisable-3d-printed-implants-and-cutting-guides-versus-non-3d-printed-standard-implants-and-cutting-guides-for-improving-outcome-in-patients-undergoing-knee-maxillofacial-o/
5. https://eunethta.eu/otca18-final-assessment-report-is-now-available/
6. https://eunethta.eu/the-ca-of-ja3-wp4-on-antibacterial-coated-sutures-versus-non-antibacterial-coated-sutures-for-the-prevention-of-abdominal-superficial-and-deep-incisional-surgical-site-infection-ssi-is-now-ava/
7. https://eunethta.eu/the-joint-assessment-on-continuous-glucose-monitoring-cgm-real-time-and-flash-glucose-monitoring-fgm-as-personal-standalone-systems-in-patients-with-diabetes-mellitus-treated-with-insuli/
8. https://eprints.aihta.at/1227/1/HTA-Projektbericht_Nr.126.pdf
9. http://doi.org/10.57598/R368C
10. https://doi.org/10.57598/R237C
11. https://doi.org/10.57598/R238C
12. https://doi.org/10.57598/R240C
13. https://doi.org/10.57598/R241C
14. https://doi.org/10.57598/R327C
15. https://doi.org/10.57598/R343C
16. https://doi.org/10.57598/R254C
17. https://doi.org/10.57598/R255C
18. https://doi.org/10.57598/R297C
19. https://doi.org/10.57598/R306C
20. https://doi.org/10.57598/R307C
21. https://doi.org/10.57598/R308C
22. https://doi.org/10.57598/R316C
23. https://doi.org/10.57598/R336C
24. https://doi.org/10.57598/R360C
25. https://doi.org/10.57598/R362C
26. https://cadth.ca/sites/default/files/ou-tr/op0530-hpv-testing-for-pcc-report.pdf
27. https://cadth.ca/sites/default/files/ou-tr/op0533-ht0024-dapt-post-pci-created-report.pdf
28. https://cadth.ca/sites/default/files/ou-tr/op0548-hcl-consolidated-report-appendices-7.0.pdf
29. https://cadth.ca/sites/default/files/pdf/ht0021_dental_amalgam_report_final.pdf
30. https://cadth.ca/sites/default/files/pdf/OP0526_Dialysis_Modalities_Science_Report.pdf
31. https://cadth.ca/sites/default/files/pdf/op0532-migs-science-report.pdf
32. https://cadth.ca/sites/default/files/pdf/TR0009_Anti-VEGF_TR_Science_Report.pdf
33. https://www.cadth.ca/sites/default/files/hta-he/HT0037-iCBT-for-Chronic-Pain.pdf
34. https://cadth.ca/sites/default/files/attachments/2021-12/OP0549-RM%20for%20Cardiac%20Conditions%20Final-meta_0.pdf
35. https://www.hiqa.ie/sites/default/files/2021-09/Advice-to-NPHET-Effectiveness-of-RADTs.pdf
36. https://www.hiqa.ie/sites/default/files/2023-09/T1D-Report-September-2023.pdf
37. https://www.hiqa.ie/sites/default/files/2023-08/Rapid%20HTA-of-flu-vaccine-50-64-general-population.pdf
38. https://www.fhi.no/en/publ/2019/Patent-foramen-ovale-closure-antiplatelet-therapy-or-anticoagulation-therapy-alone-for-management-of-cryptogenic-stroke/
39. https://www.fhi.no/en/publ/2021/Surgical-Treatment-of-Carpal-Tunnel-Syndrome/
40. http://www.iacs.es/wp-content/uploads/2019/09/13_IACS_2015_FLUIDOTERAPIA_DEF_NIPO_MOD.pdf
41. <https://icer.org/beta-thalassemia-2022/#timeline>

**Supplementary Figure 1.** Flow diagram of retrieval and selection of HTA records.

**Identification of studies via the International HTA Database**

Records identified from:

Databases (n =1215)

**Identification**

Records screened

(n = 1215)

Duplicate records excluded

(n =132 )

Reports sought for retrieval

(n = 1083)

Reports not retrieved

(n = 86)

**Screening**

Reports assessed for eligibility

(n = 997)

Reports excluded:

Awards (n =145)

Health research (n = 56)

Language (n = 41)

Resolutions (n=37)

Other (n=29)

Records included in the analysis

(n =689)

**Included**
